# Local motion governs visibility and suppression of biological motion in continuous flash suppression

**DOI:** 10.1167/jov.25.12.25

**Published:** 2025-10-27

**Authors:** William Swann, Matthew Davidson, Gabriel Clouston, David Alais

**Affiliations:** 1 University of Sydney, School of Psychology, Sydney, Australia; 2 University of Technology Sydney, Faculty of Health, Sydney, Australia; 3 University of Sydney, School of Psychology, Sydney, Australia; 4 University of Sydney, School of Psychology, Sydney, Australia

**Keywords:** continuous flash suppression, binocular rivalry, visual suppression, awareness

## Abstract

Presenting unique visual stimuli to each eye induces a dynamic perceptual state where only one image is perceived at a time, and the other is suppressed from awareness. This phenomenon, known as interocular suppression, has allowed researchers to probe the dynamics of visual awareness and unconscious processing in the visual system. A key result is that different categories of visual stimuli may not be suppressed equally, but there is still a wide debate as to whether low- or high-level visual features modulate interocular suppression. Here we quantify and compare the strength of suppression for various motion stimuli in comparison to biological motion stimuli that are rich in high-level semantic information. We employ the tracking continuous flash suppression method, which recently demonstrated uniform suppression depth for a variety of static images that varied in semantic content. The accumulative findings of our three experiments outline that suppression depth is varied not by the strength of the suppressor alone but with different low-level visual motion features, in contrast to the uniform suppression depth previously shown for static images. Notably, disrupting high-level semantic information via the inversion or rotation of biological motion did not alter suppression depth. Ultimately, our data support the dependency of suppression depth on local motion information, further supporting the low-level local-precedence hypothesis of interocular suppression.

## Introduction

Understanding visual processing outside of awareness is notoriously challenging (Holender, 1986; Schmidt, Belopolsky, & Theeuwes, 2015), and interocular rivalry/suppression has emerged as a common approach to study it. Interocular rivalry is induced by presenting incompatible images simultaneously to each eye, resulting in the conscious awareness of one image at a time (with the other suppressed) in a dynamically alternating percept. Probing sensitivity to the suppressed image in either the binocular rivalry (BR) or continuous flash suppression (CFS) paradigms has allowed researchers to better understand the functional and neural correlates of visual processing outside of visual awareness (Blake, 1989; Stein, 2019).

CFS is a form of interocular rivalry that produces extended periods of invisibility of a target image in one eye, suppressed from awareness by a high-contrast dynamic mask in the other. The breaking continuous flash suppression (bCFS) method has become widely adopted in the study of unconscious processing (e.g., Jiang, Costello, & He, 2007; Stein, Hebart, & Sterzer, 2011) and involves measuring the time it takes for a weak, low-contrast image to break into awareness. A key claim is that bCFS breakthrough times are an index of visual processing outside of awareness due to semantic content or saliency (reviewed in Yang, Brascamp, Kang, & Blake, 2014). For example, faster breakthrough times have been found for faces compared to nonface objects (Jiang et al., 2007; Yang, Zald, & Blake, 2007), and these are interpreted as evidence of preferential or prioritized unconscious processing of ecologically relevant stimuli. Similarly, arguments are made for breakthrough times for familiar words compared to unfamiliar words (Jiang et al., 2007; Yang et al., 2007) and complex scenes with incongruent versus congruent imagery (Mudrik, Breska, Lamy, & Deouell, 2011). An alternative view is that such differences are driven by low-level features such as contrast energy and spatial frequency in the target image (e.g. Alais, Coorey, Blake, & Davidson, 2024; Gayet, Van der Stigchel, & Paffen, 2014; Moors, Boelens, Van Overwalle, & Wagemans, 2016; Moors, Hesselmann, Wagemans, & van Ee, 2017; Stuit, Paffen, & Van der Stigchel, 2023).

This discrepancy remains a major controversy in the field (Stein, 2019), and the use of breakthrough times alone in the standard bCFS paradigm may be insufficient to effectively measure unconscious processing (Alais et al., 2024; Stein, 2019). More specifically, any conclusions made from reaction times in bCFS may suffer from the issue of unidirectionality, whereby thresholds are only collected when breaking from suppression into awareness, and a “common baseline” of equal invisibility during suppression is assumed so that faster breakthrough times are taken to indicate expedited or prioritized visual processing outside of awareness. Crucially, however, thresholds in the other direction are never measured (i.e., a visible image succumbing to suppression), and neither is the difference between breakthrough and suppression, which would indicate the strength of suppression of a given image—a measure commonly used in binocular rivalry to quantity suppression strength for a given image (Lunghi & Alais, 2015; Nguyen, Freeman, & Alais, 2003; Paffen, Alais, & Verstraten, 2005).

**Figure 1.**
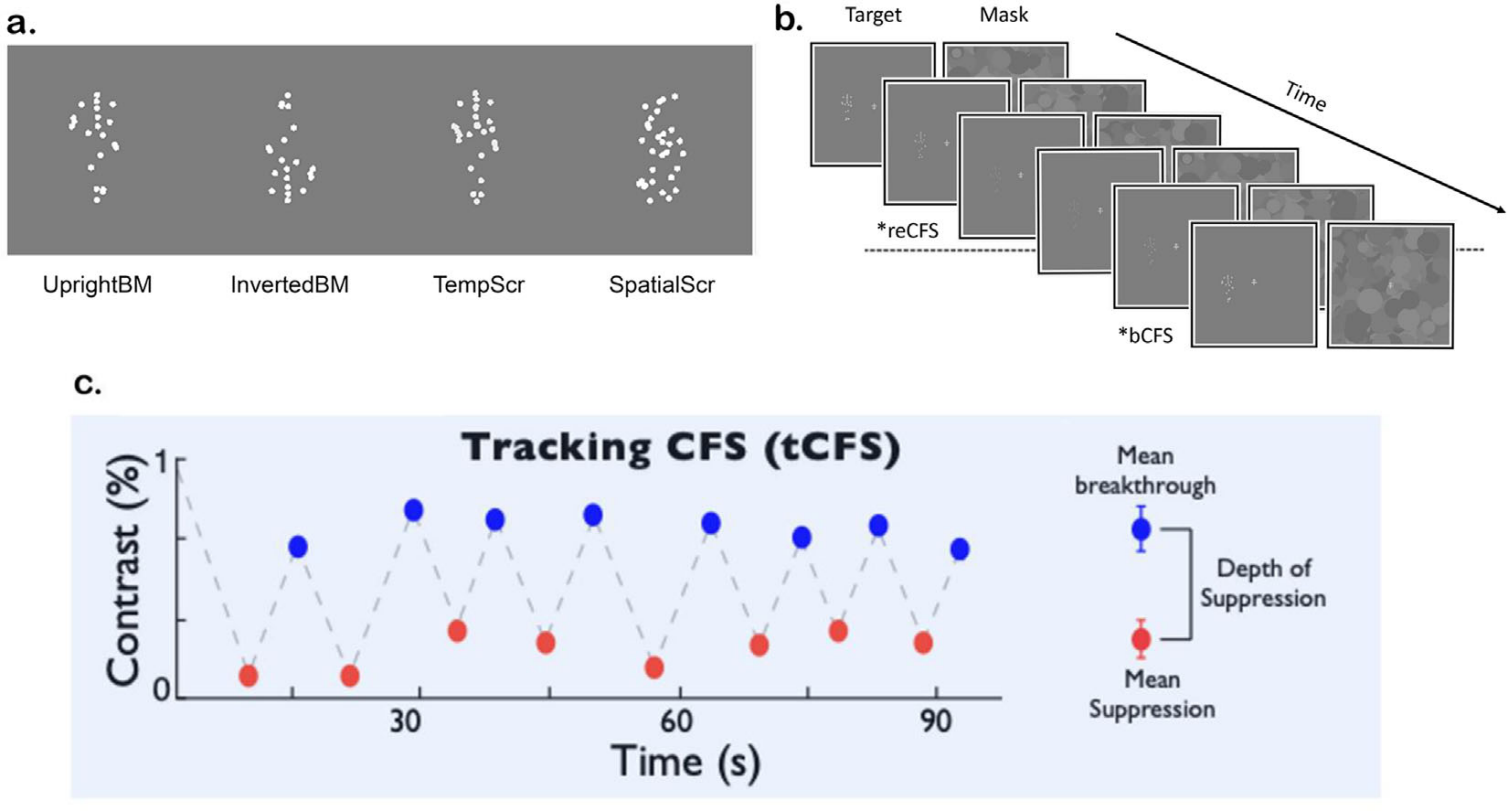
Task design and example stimuli in Experiment 1. (**a**) Example stimuli used. Note that the same biological motion stimuli were used to conserve the speed and local motion for each of the 31 dots, where inversion, temporal-phase scrambling, or spatial scrambling was applied to alter global form structure. (**b**) Example portion of a tCFS trial. A dynamic mask updating at 12 Hz and a collection of moving dots increasing and decreasing in contrast (received by the other eye). A trial begins with the target at maximum contrast and decreases in contrast on a log scale (−0.7 dB per frame). Participants respond when the moving stimulus is suppressed by the mask (reCFS threshold measurement), after which the target increases in contrast until breakthrough (bCFS measurement). *The stimulus received by the right eye is present on the left in this figure, for ease of viewing over time. (**c**) Example data from a recorded trial (showing the zigzag alternating thresholds, one condition). Suppression depth is calculated as the difference between bCFS and reCFS thresholds and expressed in decibels.

To amend these issues, a novel “tracking-CFS” (tCFS) paradigm was developed (Alais et al., 2024). This method measures contrast thresholds for a target that alternately rises in contrast until breakthrough into awareness (the bCFS threshold) and then declines in contrast until it becomes suppressed again (the resuppression threshold: reCFS), continuing in this cycle for several repetitions (see Figure 1). Using these measures, suppression depth can then be calculated as the difference between the thresholds, as commonly used in binocular rivalry studies (Alais & Melcher, 2007; Nguyen et al., 2003). As contrast is the main driver of neural response in the early visual cortex, measuring suppression depth as a contrast difference relates suppression more directly to how strongly a suppressed image is represented in the cortex.

Alais and colleagues (2024) introduced tCFS and demonstrated that bCFS thresholds do vary between image categories, as previous research has shown (Jiang et al., 2007; Kouider & Dehaene, 2007; Mudrik, Faivre, & Koch, 2014). However, they also found that when using the bCFS threshold as a starting point, the reduction in contrast needed to re-suppress an image was a uniform decrement across all the images they tested, regardless of differences in complexity and saliency (e.g., faces vs. gratings). Thus, suppression depth was uniform, even though bCFS levels varied, and this was interpreted as evidence for a single suppression mechanism early in the visual processing system, as commonly hypothesized in the binocular rivalry literature (Alais & Blake, 2014; Johansson, 1973). Finding a uniform suppression contradicts a large body of literature advocating for the preferential processing of meaningful objects outside of visual awareness, such as faces (Blake & Logothetis, 2002; Hedger, Adams, & Garner, 2015; Mudrik et al., 2014). A potential explanation suggested by proponents of this contradictory “top-down” theory is that faces—especially fearful faces—may be processed via a pathway that bypasses the early visual cortex (Duarte, Abreu, & Castelo-Branco, 2022; Tamietto et al., 2009).

It is also notable that tCFS has so far only been tested with static target images. Movement can capture attention and be ecologically relevant (e.g., biological motion), yet such stimuli have so far not been tested in the tCFS paradigm. Here, we use biological motion stimuli (Chung & Khuu, 2014; Kaunitz, Fracasso, Lingnau, & Melcher, 2013) to determine whether changes to the low- or high-level feature content of these global stimuli modulate suppression depth during tCFS. One reason biological motion is relevant is that it relies on the integration of both form and motion (Oram & Perrett, 1994), as exemplified by the point-like walkers created by Johansson (1973). Although point-light walker displays evince a salient perception of biological motion, the percept is—similar to faces—dramatically impeded when inverted (Dittrich, 1993; Pavlova & Sokolov, 2000) or rotated (Pavlova & Sokolov, 2000). As with faces, the low-level characteristics are retained, yet the global percept is not. Second, previous research suggests motion information is processed outside of awareness, as when rendered invisible by binocular rivalry (Wiesenfelder & Blake, 1991) or CFS (Kaunitz, Fracasso, & Melcher, 2011; Maruya, Watanabe, & Watanabe, 2008). In CFS, for example, a moving target presented to the suppressed eye produces a motion aftereffect (Kaunitz et al., 2011; Maruya et al., 2008). Together, this prior research indicates that features of invisible motion stimuli and the dominant mask may interact to alter suppression depth when measured using the tCFS paradigm.

The question of which types of information integration do and do not require conscious awareness remains to be definitively answered (Alais, 2012; Kouider & Dehaene, 2007). An investigation of biological and coherent motion, in the context of tCFS and suppression depth, would help to unpack inconsistencies in the literature as to whether lower- or higher-level processing is preserved during continuous flash suppression. We therefore performed three experiments to compare the suppression depth of moving targets imbued with biological motion against relating stimuli where the low-level and high-level features have been manipulated. To preview our results, we find that the determinant of suppression depth is not the strength of the suppressor alone, as altering local motion feature information (i.e., via spatial scrambling or increasing speed) has an influence on suppression depth. Notably, and in contrast, varying the high-level semantic information (i.e., via biomotion inversion) did not alter suppression depth. We interpret these results in support of the low-level, local-precedence hypothesis (Sun, Wang, Huang, Ji, & Ding, 2022) of interocular suppression.

## Experiment 1: Assessing the suppression depth of biological motion during tCFS

Experiment 1 addresses two main questions. First, is there a difference in suppression depth between upright biological motion compared to inverted biological motion? Second, how does suppression depth change when the global biological form is disrupted, either by spatial or temporal scrambling?

We have three hypotheses. First, because previous tCFS research has demonstrated a consistent suppression depth between image categories (Alais et al., 2024), there will be no significant difference in suppression depth between our conditions. Second, we hypothesize that despite this consistent suppression depth, there will be a significant difference in mean bCFS thresholds and reCFS thresholds between stimuli, as previous results have shown (Alais et al., 2024). We predict that upright biological motion will have a lower bCFS threshold than inverted biological motion, as predicted by prior research (Sun et al., 2022; Watson, Pearson, & Clifford, 2004). Similarly, we predict that manipulations of global form will increase bCFS thresholds compared to upright biological motion, as their global motion information is disturbed (Grossman & Blake, 2001; Sun et al., 2022).

### Method

#### Participants

In total, 18 participants were recruited for Experiment 1, all of whom were naive to the purpose of the study, had normal or corrected-to-normal vision, and were informed of the protocol and the anonymity of the data. All provided informed consent before proceeding. Three met the criteria for exclusion, which included the inability to maintain binocular fusion (*n* = 1), the identification of ceiling or floor effects in postprocessing (*n* = 1), and the misunderstanding of instructions (*n* = 1). The final sample (*N* = 15) had a median age of 30 years (age range, 18−68; 3 left-handed; 9 females). The study was approved by the University of Sydney Human Research Ethics Committee (HREC 2021/048).

#### Power calculations

We performed a series of power analyses using G*Power Version 3.1 (Faul, Erdfelder, Buchner, & Lang, 2009) to estimate the sample size necessary to detect a difference in suppression depth within our various repeated-measures designs. Only one previous behavioral study has used tCFS to investigate differences in suppression depth and found a very large effect size ($\eta_{p}^{2}$ = 0.89) in a 1 × 3 repeated-measures design (Experiment 3 of Alais et al., 2024). This extremely large effect size was obtained after modulating the rate of contrast change during tCFS. Here, as our manipulations are more subtle (varying target types), we adopted more conservative estimates based on a moderate effect size (Cohen's *f* = 0.3). This updated parameter requires *n* = 17, *n* = 14, and *n* = 21 for Experiments 1 through 3, respectively. In each case, we opted for larger sample sizes to account for variability in participant data and potential exclusion.

#### Apparatus and stimuli

All experiments were programmed using custom MATLAB code and displayed using MATLAB (Version 2022b) and Psychtoolbox (Version 3.0.13; Brainard, Brunt, & Speigle, 1997). The stimuli were presented on an ASUS VS247H-P LED monitor (23.6 inches, 1,920 × 1,080 pixels, 60-Hz refresh rate). A mirror stereoscope was used to partition participant's vision into separate left- and right-eye views, positioned 50 cm from the screen, with a total optical path length of 57 cm. When viewing through the stereoscope, participants were presented with a high-contrast Mondrian mask pattern (400 × 400 pixels, 7° × 7°) to their left eye and a small target stimulus (112.5 × 112.5 pixels, 2.2° × 2.2°) presented to their right eye. Two binocularly presented white squares surrounded the mask, with a fixation cross in the center (18 × 18 pixels; 0.3° × 0.3°), which both served as a fusion lock to maintain stable fusion. A chin rest was provided to minimize movement and further aid stable binocular fusion.

We used grayscale Mondrian patterns (see Figure 1b) to match our targets, as previous achromatic masks have been shown to be optimal to suppress achromatic targets (Pournaghdali & Schwartz, 2020). The Mondrian mask patterns included multiple overlapping circles (of various sizes and contrast values), which were updated at a frequency of 12 Hz and had a coded maximum contrast value of 0.1. All target stimuli were sourced from the Biomotion Toolbox (van Boxtel & Lu, 2013). Moving joint coordinates of a point-light walker in three-dimensional space (File “02_02.bvh”) were presented as 31 white moving dots (target motion boundary: 112 × 112 pixels, scale setting = 0.75 in 150-pixel subspace), with a dot diameter of 4 pixels (0.1°). The animation sequence consisted of 300 frames looped to provide the appearance of continuous walking. This same animation file of 31 white moving dots was manipulated further to create each of the four target conditions in Experiment 1, as well as to create the subsequent target conditions for Experiments 2 and 3.

Experiment 1 utilized the inbuilt inversion, temporal-phase scramble, and spatial scramble parameters of the Biomotion Toolbox to manipulate the holistic/high-level features of the animation sequence. Importantly, in each manipulation, the local dot motion vectors of the biological motion animation are preserved, but the global relationship between local features is changed. These targets were placed on a mid-gray background, with the center location of each target alternating 200 pixels (5°) to the left or right of the fixation cross between trials (Figure 1). Each target's contrast was ramped up or down by scaling its standard contrast within a range of 0.02 to 0.10 to capture both bCFS and reCFS thresholds during tCFS (Alais et al., 2024). Importantly, contrast increased linearly in decibel units (i.e., a logarithmic scale) so that contrast change was perceptually linearized. This made the perceived changes in contrast effectively linear, given the visual system's logarithmic contrast response function (De Valois & De Valois, 1991). Minimum (“0.02”) and maximum (“0.10”) contrast were thus −33.98 and 0 dB, respectively, and contrast steps were 0.07 dB per frame. The mask contrast was held at 0 dB.

#### Design

Experiment 1 used a 2 (bCFS, reCFS) × 4 (upright, inverted, temporally scrambled, or spatially scrambled biomotion) repeated-measures design. A single tCFS trial consisted of six bCFS and six reCFS responses to a single target stimulus ramping up or down in contrast strength. Each motion target was presented once per block in a new randomized order (as per a 4-trial × 8-block design). This resulted in 12 responses per trial (6 bCFS, 6 reCFS), repeated eight times for each of the four conditions (per participant). Total mean bCFS and reCFS values were calculated for each of the four targets in Experiment 1, and a two-way repeated-measures analysis of variance (ANOVA) was conducted. The whole experiment took between 20 and 40 minutes (depending on the number of breaks and the participants’ suppression depth).

#### Procedure

First, participants read the Participant Information Sheet, had the opportunity to ask questions, and provided written informed consent before we collected their data. Participant data involving their age, gender, left/right-handedness, and left/eye dominance were collected before explaining the experimental conditions. The stereoscope was then calibrated (via fusion of stimulus borders and adjusting the mirrors) for each participant, and then each underwent practice trials until they understood the instructions for tCFS (∼ two trials on average). During the practice session, we emphasized the importance of maintaining focus on the central cross of the display screen at all times and to try to refrain from blinking until the end of a trial (after 12 clicks). The dependent variable was the contrast of the target at the moment a click response was recorded (indicating either breaking or reentering of suppression).

#### Data analysis

The bCFS and reCFS thresholds were averaged to obtain a mean bCFS and reCFS per condition per participant. Data analysis was performed in MATLAB (Version R2022b) and SPSS/JASP (Version 0.17.3). For analysis and visualization, all contrast thresholds are expressed in decibel units (dB). Along with repeated-measures ANOVA statistical analyses and Bonferroni-corrected post hoc comparisons, we also performed a Bayesian model comparison to quantify evidence for and against the null, using Bayesian repeated-measures ANOVAs (uninformed prior with equal weight to all models) in JASP. Reported are Bayes factors (*B*) for main effects of interest (e.g., effect of biomotion type on suppression depth), as evidence in favor of the alternatives compared to the null model (BF_10_ = *B*) or evidence in favor of the null model over the alternative hypothesis (BF_01_ = *B*). Following the guidelines recommended in Dienes (2021), *B* values greater than 3 indicate moderate evidence for the model under comparison, H1 over H0. *B* values residing between 1/3 and 3 are interpreted as weak evidence, or an insensitivity in the data to distinguish between the models.

### Results of Experiment 1: The effect of motion targets on suppression depth

**Figure 2.**
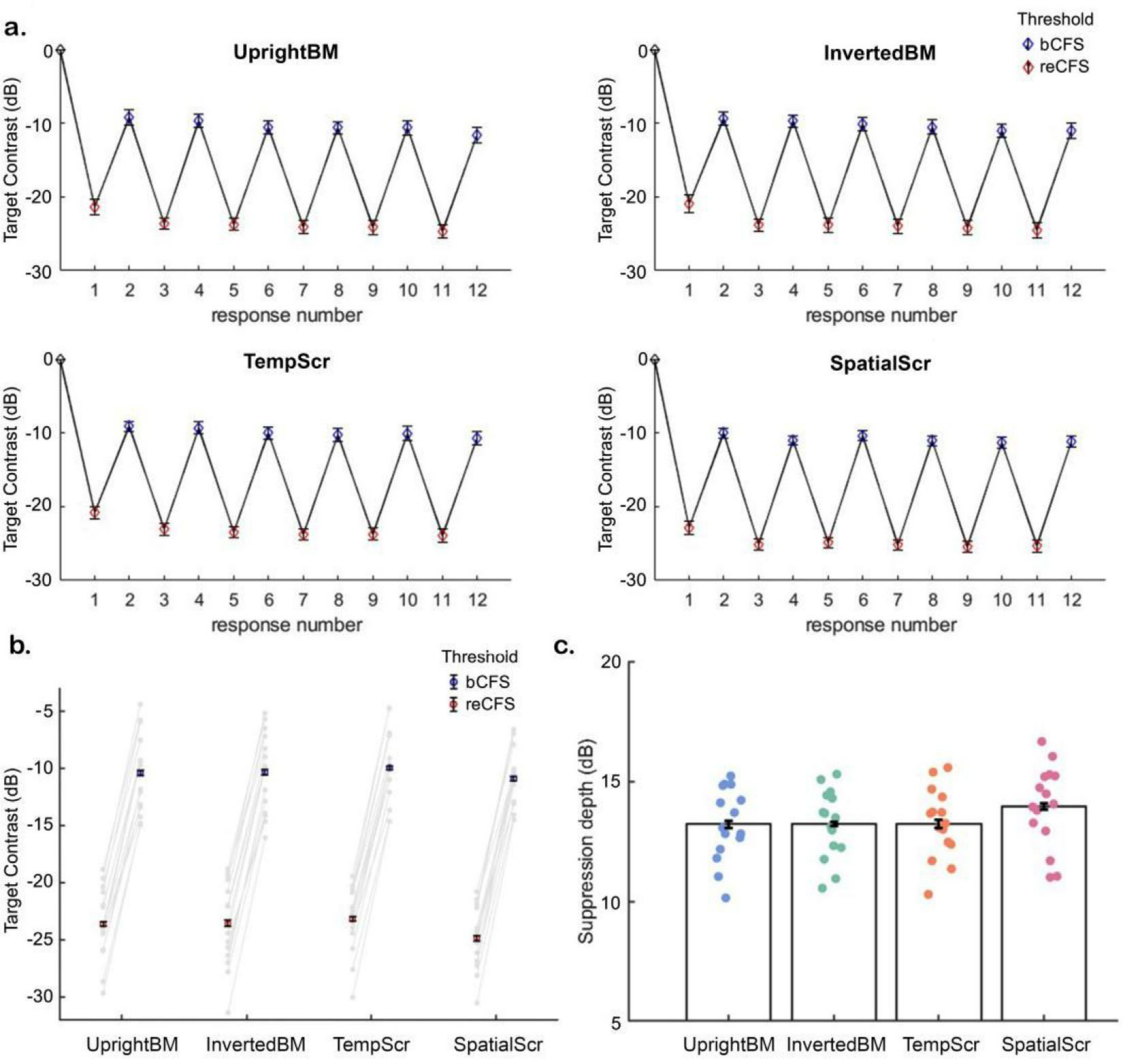
Results from Experiment 1. (**a**) Mean bCFS and reCFS thresholds per response (1−12) for each biomotion type*.* The first point at maximum contrast (0 dB) represents the starting contrast for each trial, followed by the first response (reCFS). Error bars represent ±1 *SEM* corrected for within-participant comparisons (Cousineau, 2005). (**b**) A significant difference between bCFS and reCFS thresholds was recorded for all four biomotion conditions. Gray dots and lines connect individual participant data within each condition. (**c**) Suppression depth for each of the biomotion conditions. Suppression depth is the difference between the bCFS and reCFS thresholds. Colored dots indicate participant-level data. Error bars in panels b and c represent ±1 *SEM* corrected for within-participant comparisons (Cousineau, 2005). Note that error bars in panel b are occluded by individual markers.

A 2 × 4 within-subjects, repeated-measures ANOVA compared both contrast thresholds (bCFS, reCFS) across four motion targets (upright, inverted, temporally scrambled, or spatially scrambled biomotion). There was a significant main effect of contrast thresholds, indicating a substantial difference between bCFS (*M* = −10.4 dB, *SD* = 3.0) and reCFS (*M* = −23.8 dB, *SD* = 3.1) values (*F*(1, 14) = 1,302.1, *p* < 0.001, $\eta_{p}^{2}$ = 0.99; Figure 2). There was also a significant main effect of biomotion target (*F*(3, 42) = 5.10, *p* = 0.004, $\eta_{p}^{2}$ = 0.27), as well as a significant interaction effect observed between threshold and biomotion target (*F*(3, 42) = 5.089, *p* = 0.004, $\eta_{p}^{2}$ = 0.27). This interaction indicates that suppression depth varied between biomotion types. Figure 2b provides a summary of these results.

To perform post hoc comparisons on suppression depth, we repeated our analysis now using a 1 × 4 repeated-measures ANOVA (removing bCFS/reCFS thresholds and focusing on suppression depth alone across four motion conditions). Post hoc comparisons using Bonferroni correction revealed a significantly greater mean suppression depth for spatially scrambled biomotion (*M* = 13.95 dB, *SD* = 1.72) than temporally scrambled biomotion (*M* = 13.24 dB, *SD* = 1.46; *t*(14) = 3.81, *p* = 0.003, Cohen's *d* = −0.414), but not for upright biomotion (*M* = 13.22 dB, *SD* = 1.51, *t*(14) = 2.40, *p* = 0.126) or inverted biomotion (*M* = 13.23 dB, *SD* = 1.44, *t*(14) = 2.63, *p* = 0.071). Additionally, we quantified the evidence for the observed effect on suppression depth in Experiment 1 with a subsequent Bayesian model comparison. A Bayesian repeated-measures ANOVA (1 × 4; suppression depth × target type) found that the variation in biomotion type was a probable model to explain observed differences in suppression depth (BF_10_ = 9.51). This indicates that the data support that biomotion type was better at predicting suppression depth than the null model.

### The effect of motion targets on bCFS or reCFS in isolation

Looking at the traditional measure of bCFS thresholds alone, we again repeated our analysis using two 1 × 4 repeated-measures ANOVAs, focusing either on bCFS or reCFS thresholds in isolation. There were no significant differences in bCFS between motion types after Bonferroni correction (all *p*s > 0.05). However, a significant difference was present between reCFS measures, which helps to explain the difference in suppression depth between motion types. Here, the mean reCFS for spatially scrambled motion was significantly lower than for temporally scrambled motion (*t*(14) = −4.60, *p* < 0.001, Cohen's *d* = −0.529) and the inverted biomotion condition (*t*(14) = −3.51, *p* = 0.026, Cohen's *d* = −0.403), but not for upright biomotion (*p* = 0.05).

### Experiment 1 discussion

Findings from Experiment 1 reject our main hypothesis that there would be consistent suppression depths between biomotion types, instead indicating that suppression is not controlled by the strength of the suppressor alone but also by motion type. This differs from previous research using tCFS, which found consistent suppression depth between different static images (Alais et al., 2024). In particular, there was a significant increase in suppression depth for the spatially scrambled motion but no difference in suppression depth when comparing upright and inverted biological motion. This latter finding is in opposition to other studies where a significant inversion effect for biological motion was found in the context of bCFS (Sun et al., 2022), although this study used breakthrough times and not contrast thresholds (within the binocular rivalry paradigm; Watson et al., 2004). The absence of this effect in the present study may suggest that CFS suppression operates on local motion information, as local information is relatively conserved (compared to high-level information) when biological motion is inverted (Pavlova & Sokolov, 2000). Notably, temporally scrambled biomotion—which also retains the trajectories of normal biomotion but starts each point at a different temporal position—did not statistically differ from the upright or inverted biological motion.

Why, then, was suppression depth different when the dots were scrambled in their spatial relationship and local dot motion was also conserved? Subsequent analysis attributed the source of this difference to a lower reCFS threshold for spatial scrambled motion without an equivalent change in bCFS threshold. These findings highlight the importance of considering suppression thresholds in CFS (i.e., reCFS), as bCFS thresholds alone do not necessarily capture all that is relevant about a given condition. When taking into account both of these measures, a change in suppression depth was supported, which would have been absent if the focus had been on bCFS thresholds alone.

One possible account for this change in reCFS threshold is that the spatially scrambled stimuli occupied an enlarged portion of the visual field compared to the alternatives. Indeed, on review of the stimuli, the spatially scrambled motion had dots presented over more of the two-dimensional space owing to the random selection of starting positions within the bounds of the overall animation display when implemented in the biomotion toolbox. This is in contrast to the stimuli in the other biomotion conditions that had many dot positions in close proximity (or even overlapping) in areas such as the head, neck, shoulders, and hips, with a highly correlated pattern and direction of movement between dots in the non–spatially scrambled biomotion conditions. Therefore, of the 31 dots, several of them were overlapping, making the points occupy less space and seem less numerous. Future research may use fewer dots or an alternative spatial scrambling technique to resolve this issue.

Interestingly, others have found that when scrambling biological motion, there is no a priori structure (specific locations or configurations of local information) that influences priority access (Sun et al., 2022). This suggests that the greater number of dots (or the interaction between the number of dots across a greater space) is the likely cause of a reduced reCFS threshold (and larger suppression depth) in our experiment.

Our next experiment sought to verify and replicate our null result from Experiment 1 of no inversion effect on bCFS thresholds, while investigating low-level visual features in further detail. To investigate further the influence of low-level visual features on suppression depth, we next addressed how changes in mask contrast alter suppression depth measures.

## Experiment 2: Testing the effect of mask contrast on suppression depth

The results of Experiment 1 demonstrated that features of the suppressed target and dominant mask interacted to determine suppression depth. More specifically, spatially scrambled biomotion stimuli required a larger change in contrast to enter and exit awareness compared to the other biological motion conditions, which retained the local spatial relationships between dots and occupied a relatively smaller visual region. Experiment 2 assessed this joint contribution between target and mask features in a novel way, by examining how changes to the strength (i.e., contrast) of the mask altered tCFS thresholds. While investigating the effect of mask contrast, we additionally sought to self-replicate the null effect of biological motion inversion, which stands counter to previous research (Sun et al., 2022). We have two main questions for Experiment 2. First, does suppression depth in the tCFS paradigm stay the same or vary with different mask contrasts? Second, can we replicate the null result regarding biological motion inversion, or is the absence of an inversion effect dependent on mask contrast?

Regarding the first question, previous research has investigated the influence of reducing mask contrast and found a reduction in suppression, as noted by reduced bCFS breakthrough times (Han, Lukaszewski, & Alais, 2019). However, using the tCFS procedure, it is possible to quantify both bCFS and reCFS thresholds, and it is currently unknown how changes in mask contrast may affect reCFS thresholds or whether the effect of mask contrast may interact with each threshold type. Indeed, in Experiment 1, motion type uniquely impacted upon reCFS thresholds, opening the possibility that similar asymmetries may be captured when assessing the contribution of mask contrast during tCFS. This is important as it could specify how the mechanism of suppression in CFS is modulated by mask contrast, potentially modulating suppression depth through a change in bCFS thresholds, reCFS thresholds, or both. First, we hypothesize that when reducing mask contrast strength, we will observe a reduction in bCFS thresholds. This is in alignment with the faster breakthrough times previously reported for reduced mask contrasts in previous literature (Han et al., 2019).

Second, as previous work has demonstrated that suppression is jointly determined by the composition of the target and the competing mask (Yang & Blake, 2012), we predicted that increasing or decreasing mask contrast would modulate the strength of interocular suppression and consequent suppression depth. This follows our previous work, which altered suppression depth by modulating the strength of interocular competition via changes to the rate of adaptation (Alais et al., 2024). Here, we sought to extend this evidence by modulating interocular competition using changes to mask characteristics. Specifically, we hypothesize that reduced mask strength will lower interocular competition, requiring a smaller change in contrast for a stimulus to enter or exit awareness, which will be quantified as a decrease in suppression depth during tCFS.

Third, we aim to replicate the null effect of inversion found in Experiment 1, as well as identify whether there is any interaction effect between mask contrast strength and biomotion orientation (upright or inverted). To test this, we will use the same biological motion stimuli as in Experiment 1, with the inclusion of masks at low, medium, or high contrast.

### Method

#### Participants

We recruited 23 first- and second-year psychology students for Experiment 2, of whom 3 met the criteria for exclusion: inability to maintain binocular fusion (*n* = 1), the identification of ceiling or floor effects in postprocessing (*n* = 1), and the misunderstanding of instructions (*n* = 1). The final sample (*N* = 20) had a median age of 23 years (age range, 19−32; 2 left-handed; 12 females). Participants were reimbursed with course credit. This study was approved by the University of Sydney Human Research Ethics Committee (HREC 2021/048).

#### Procedure, apparatus, and stimuli

The procedure, apparatus, and biomotion stimuli were identical to Experiment 1. Experiment 2 used three levels of mask strength, manipulated by varying the root mean square (RMS) contrast (i.e., the standard deviation of the pixel intensities) of the mask contrast over three levels: 0.07, 0.1, or 0.13 (where 0.1 matches Experiment 1). As in Experiment 1, Experiment 2 implemented the “Inversion” function using the biomotion toolbox (van Boxtel & Lu, 2013).

#### Design

Experiment 2 used a 2 (bCFS, reCFS) × 3 (low, medium, and high mask contrast) × 2 (upright vs. inverted) repeated-measures design. Following the same protocol as in Experiment 1, each motion target was presented once per block (i.e., a 6-trial × 8-block design, with a new randomized order in each block), and participants clicked a mouse to indicate when a visible target became suppressed (reCFS) or when an invisible target broke through into visibility (bCFS). The whole experiment took between 30 and 50 minutes.

### Results of Experiment 2: The effect of orientation and mask contrast on suppression depth

**Figure 3.**
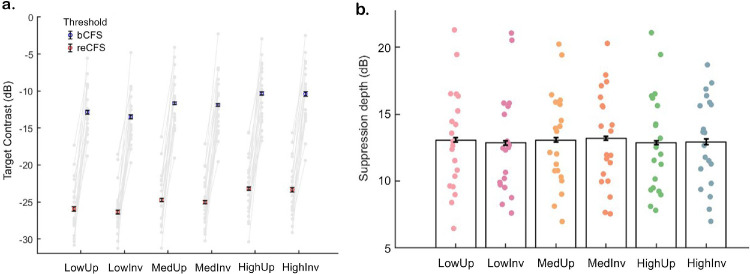
Suppression depth is not affected by mask contrast or the inversion of biological motion. (**a**) Displays the bCFS and reCFS thresholds for each condition (*N* = 23), which increased in tandem with increases in mask contrast. Gray dots and lines connect individual data per condition. (**b**) Displays suppression depth, which did not significantly differ with changes in mask contrast. Individual dots represent participant-level data. Error bars in panels a and b represent ±1 *SEM* corrected for within-participant comparisons (Cousineau, 2005) and in panel a are occluded by markers.

A 2 × 3 × 2 repeated-measures ANOVA compared both thresholds (bCFS, reCFS) across three mask contrast conditions (low, medium, and high) and two orientations (upright and inverted biomotion). There was a significant main effect of threshold type (*F*(1, 19) = 264.6, *p* < 0.001, $\eta_{p}^{2}$ = 0.93; Figure 3a). There was also a significant main effect of mask contrast (*F*(2, 38) = 36.23, *p* < 0.001, $\eta_{p}^{2}$ = 0.66). However, there was no significant main effect of inversion (*p* = 0.131). There were also no interaction effects between thresholds, mask contrast, and orientation observed (all *p*s > 0.05).

Post hoc comparisons were conducted after repeating our analysis using a 1 × 6 repeated-measures ANOVA that removed bCFS/reCFS labels (suppression depth × 6 motion targets). Figure 3b highlights the results from this analysis and that mean suppression depth (*M* = 13.0 dB, *SD* = 3.6) is highly conserved (*F*(5, 95) = 0.24, *p* = 0.945, $\eta_{p}^{2}$ = 0.01) across the six conditions (LowUp: *M* = 13.07 dB, *SD* = 3.79; LowInv: *M* = 12.85 dB, *SD* = 3.77; MedUp: *M* = 13.07 dB, *SD* = 3.57; MedInv: *M* = 13.18 dB, *SD* = 3.61; HighUp: *M* = 12.87 dB, *SD* = 3.78; HighInv: *M* = 12.93 dB, *SD* = 3.36). Experiment 2 therefore indicates that the relationship between bCFS and reCFS is invariant across mask type and biomotion orientation.

We quantified the evidence for this null effect on suppression depth with a subsequent Bayesian model comparison. A Bayesian repeated-measures ANOVA (2 × 3; target orientation × mask contrast) found moderate evidence for the null model over the main effect of mask contrast (BF_01_ = 4.13) and for the null model over an effect of target orientation (BF_01_ = 4.77), with no interaction (BF_01_ = 84.62). Overall, these results indicate neither target orientation nor mask contrast modulates suppression depth.

#### The effect of orientation and mask contrast on bCFS or reCFS in isolation

We repeated our analysis with a series of 1 × 6 repeated-measures ANOVAs to focus on either bCFS or reCFS thresholds alone and enable post hoc comparisons with Bonferroni corrections. For both, a significant main effect of testing condition (mask contrast and orientation) was found (bCFS; *F*(5, 19) = 21.37, *p* < 0.001, $\eta_{p}^{2}$ = 0.53; reCFS; *F*(5, 19) = 21.79, *p* < 0.001, $\eta_{p}^{2}$ = 0.53).

Post hoc analysis highlighted significant differences between each mask contrast level, where lowering of mask contrast led to a consistent lowering of bCFS and reCFS values (e.g., bCFS Upright; low vs. medium: *t*(19) = −3.10, *p* = 0.038, Cohen's *d* = −0.31, medium vs. high: *t*(19) = −3.42, *p* = 0.016, Cohen's *d* = −0.34; reCFS Upright; low vs. medium: *t*(19) = −3.06, *p* = 0.043, Cohen's *d* = −0.33, medium vs. high *t*(19) = −3.87, *p* = 0.003, Cohen's *d* = −0.41; Figure 3a).

### Experiment 2 discussion

Experiment 2 identified consistent suppression depths across three levels of mask contrast strength (low, medium, and high) and no difference based on the orientation of the biological motion stimuli (Upright vs. Inverted). This rejected our first hypothesis of a reduced suppression depth with reduced increments of mask contrast strength. However, confirming previous research (Han et al., 2019), we found a significant reduction in bCFS measures with reduced mask contrast.

This latter result can be explained by understanding that the mask is responsible for the phenomenon of visual suppression. Therefore, presenting a lower mask contrast to one eye will cause a relatively weaker suppression and lower breakthrough thresholds for the target presented to the opposing eye. However, the suppression depth (the contrast difference between bCFS and reCFS) was not affected by changes in mask contrast strength. As suppression depth was uniform in logarithmic units (dB), which highlights that the mechanism controlling the interocular conflict exerts a constant proportion of suppression and is likely to be a single process, most likely early in visual cortical processing, where left and right eyes are combined. Notably, this pattern of results is consistent with prior work on binocular rivalry, wherein the competing excitation and inhibition of monocular neurons in V1 were proposed to govern competing periods of dominance and suppression (Polonsky, Blake, Braun, & Heeger, 2000; Tong & Engel, 2001).

We additionally replicated the results of Experiment 1 by showing no significant difference in bCFS or reCFS thresholds when comparing upright and inverted walkers. This provides complementary evidence, alongside Experiment 1, that there is indeed no biological motion inversion effect on bCFS or suppression depth, again in opposition to the inversion effect shown by others using bCFS breakthrough times (Sun et al., 2022). Suppression depth was also conserved between separate samples of participants (e.g., Experiment 1, inverted biomotion: *M* = 13.2 dB, *SD* = 1.4; Experiment 2, medium mask inverted biomotion: *M* = 13.2 dB, *SD* = 3.6).

## Experiment 3: The influence of biomotion speed and optic flow on suppression depth

Experiments 1 and 2 have demonstrated that suppression depth is resistant to violations of semantic meaning, as no differences were found between upright and inverted biological motion. One explanation for this null result could be that each walking figure was oriented front-on, walking toward the viewer. Previous research has indicated that certain poses and orientations are recognized more readily than others (e.g., upright vs. 90° inverted rotation vs. 180° inverted rotation; Shipley, 2003); therefore, perhaps the walking figure was not well detected by the participants. Indeed, anecdotal reports by participants in Experiments 1 and 2 indicated that some were aware of the biological motion, and others were not. Perhaps the influence of biological motion on tCFS would be better captured when walkers are presented side-on, where the speed of the dots may be more noticeable and have a greater effect. To address these questions, Experiment 3 employed the same biomotion stimuli from Experiments 1 and 2, now rotated 90° around the vertical axis (walking left to right at a fixed position). As greater speeds of motion are more salient and resistant to adaptation (Ananyev, Penney, & Hsieh, 2017), we also presented the figure at three different speeds (static, normal, and double speed). As our previous work has indicated that suppression depth increases with reduced rates of adaptation (Alais et al., 2024), we hypothesized that the variations in dot speed could alter adaptation mechanisms and similarly affect tCFS thresholds.

A final motivation of ours was to expand our motion types to include an investigation of coherent optic flow. Previous work has demonstrated significant differences in suppression when comparing coherent dot motion to dynamic patterns (Chung & Khuu, 2014), as well as when comparing radially expanding optic flow to random trajectories (Kaunitz et al., 2013). Indeed, Kaunitz et al. (2013) found a significant difference in detection performance between radial motion and random walk patterns, yet no differences between biological motion and a scrambled alternative. Thus, tCFS may similarly capture a difference in suppression depth when comparing expanding/contracting optic flow to a scrambled alternative as evidence of processing motion form outside of awareness.

Experiment 3 addresses these questions: First, are tCFS thresholds modulated when dot motion occurs at different speeds for a profile-oriented walker? Second, do tCFS thresholds differ substantially between biological motion, radially expanding and contracting optic flow, and a scrambled alternative? The results of these manipulations will further help to understand whether low-level information (e.g., the speed of dots) or high-level information (e.g., the type of coherent motion) affects suppression depth.

### Method

#### Participants

For Experiment 3, we recruited 30 first- and second-year psychology students, of whom 5 met the criteria for exclusion: inability to maintain binocular fusion (*n* = 1), the identification of ceiling or floor effects in postprocessing (*n* = 2), and the misunderstanding of instructions (*n* = 2). The final sample (*N* = 25) had a median age of 23 years (age range, 18−42; 3 left-handed; 14 females). Participants were reimbursed with course credit.

This study has been approved by the University of Sydney Human Research Ethics Committee (HREC 2021/048). Power calculations were the same as Experiment 1.

#### Procedure, apparatus, and stimuli

The procedure, apparatus, and source of biomotion stimuli were identical to Experiments 1 and 2. Experiment 3 involved rotation of the walker by 90° around the vertical axis, to be seen from a side-profile viewpoint. The normal walker moved with a frequency of 1.05 steps per second (∼ 1 Hz). We also designed coherent and temporally scrambled optic flow motion targets that matched this frequency. The fast walker condition displayed the identical biomotion sequence at twice the normal frame rate. A static frame of the walker at maximum stride (frame index = 40) was used for the static stimuli and for the starting coordinates for coherent and temporally scrambled optic flow.

Using the point coordinates of the static frame as the starting positions, coherent motion required converting the Cartesian coordinates of the dots into polar coordinates. The following equation was then used to generate coherent optic flow, which progressed radially relative to the central starting point of each dot, with a sinusoidal magnitude:

$$\begin{array}{ccc} & & \;r=r+\left(0.2*r*\mathrm{amp}*\sin\left(2*\mathrm{pi}*t/\mathrm{modperiod}\right)\right) \end{array}$$

Note: The local speed varies with the distance from the central point, as with optic flow (where *r* = radius, amp = amplitude, and modperiod is coded to induce a uniform starting point in the phase per dot).

For temporally scrambled optic flow, the identical motion for each dot was used, but at a random phase, using

$$\begin{array}{ccc} & & \;r=r+(0.2*r*\text{amp}*\text{sin}(2*\text{pi}*t/\text{randOffsets})) \end{array}$$

Note: randOffsets was coded to randomize the starting point in the phase per dot.

**Figure 4.**
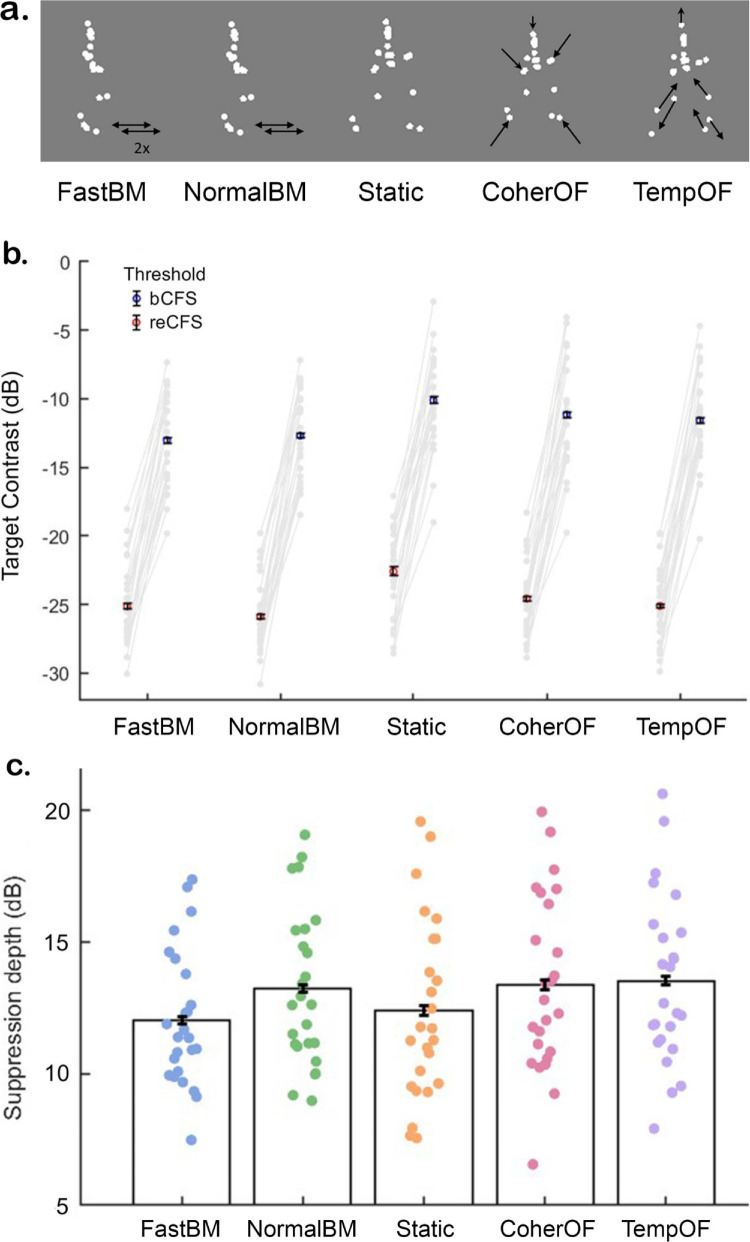
Motion type significantly impacts suppression depth. (a) All stimuli were constructed from the same biomotion stimuli used in the previous two experiments but were rotated by 90° around the vertical so that they appeared side-on to the viewer. (**b**) bCFS and reCFS thresholds were both impacted by variations in motion type. Note that error bars are occluded by individual markers. Gray dots and lines connect individual data per condition. (**c**) A significant reduction in suppression depth was observed for fast relative to normal biomotion, where normal, coherent, and temporally scrambled optic flow had consistent suppression depths. Here, normal, coherent, and temporally scrambled optic flow had significantly deeper suppression than fast biomotion and the static stimulus. Error bars represent ±1 *SEM* corrected for within-participant comparisons (Cousineau, 2005).

Schematic examples of each stimulus are given in Figure 4a and later abbreviated as FastBM, NormalBM, Static, CoherentOF, and TempOF, respectively.

#### Design

Experiment 3 used a 2 (bCFS, reCFS) × 5 (Fast BM, Normal BM, Static, CoherOF, & TempOF motion) repeated-measures design. Following the same protocol as Experiments 1 and 2, each motion target was presented once per block (i.e., a 5-trial × 8-block design). The whole experiment took between 20 and 40 minutes.

### Results of Experiment 3: The effect of biomotion speed and optic flow on suppression depth

A 2 × 5 repeated-measures ANOVA compared both thresholds (bCFS and reCFS) across five motion types (Fast BM, Normal BM, Static, CoherOF, and TempOF). There was a significant main effect of threshold (bCFS vs. reCFS; *F*(1, 24) = 456.8, *p* < 0.001, $\eta_{p}^{2}$ = 0.95). There was also a significant main effect of motion type (*F*(4, 96) = 28.02, *p* < 0.001, $\eta_{p}^{2}$ = 0.54). Additionally, there was a significant interaction effect observed between threshold and motion type (*F*(4, 96) = 14.48, *p* < 0.001, $\eta_{p}^{2}$ = 0.38), indicating the presence of varied suppression depths between motion types. These results are plotted in Figure 4b.

Additionally, we quantified the evidence for an effect on suppression depth (Figure 4c) with a Bayesian model comparison. A Bayesian repeated-measures ANOVA (1 × 5, suppression depth × motion type) found that the best model to explain suppression depth included the main effect of motion type (BF_10_ = 3.594 × 10^6^).

Following this 1 × 5 repeated-measures ANOVA, post hoc comparisons revealed significant differences in mean suppression depth between conditions. Specifically, FastBM (*M* = 12.11 dB, *SD* = 2.67) had a significantly smaller suppression depth than Normal speed BM (*M* = 13.19 dB, *SD* = 2.95; *t*(24) = −4.69, *p* < 0.001, Cohen's *d* = −0.35), CoherOF (*M* = 13.42, *SD* = 3.30 dB; *t*(24) = −5.68, *p* < 0.001, Cohen's *d* = −0.42), and TempOF motion (*M* = 13.53 dB, *SD* = 3.18; *t*(24) = −6.16, *p* < 0.001, Cohen's *d* = −0.46) but not Static (*M* = 12.48 dB, *SD* = 2.48). Additionally, the Static target produced a significantly smaller suppression depth than NormalBM (*t*(24) = −3.07, *p* = 0.028, Cohen's *d* = −0.23), CoherOF (*t*(24) = −4.06, *p* < 0.001, Cohen's *d* = −0.30), and TempOF motion (*t*(24) = −4.54, *p* < 0.001, Cohen's *d* = −0.34). There were no significant differences in suppression depth between NormalBM, CoherOF, and TempOF. In summary, fast biomotion and static biomotion had lower suppression depths than the other conditions but were not different from each other. A summary of these significant differences is displayed in Figure 4c.

#### The effect of biomotion speed and optic flow on bCFS or reCFS in isolation

We repeated our analysis using a series of 1 × 5 repeated-measures ANOVAs to focus on either bCFS or reCFS thresholds alone and perform post hoc comparisons. For both, a significant main effect of motion type was found (bCFS; *F*(1, 24) = 25.36, *p* < 0.001, $\eta_{p}^{2}$ = 0.51; reCFS; *F*(1, 24) = 27.43, *p* < 0.001, $\eta_{p}^{2}$ = 0.53).

bCFS was significantly lower for FastBM than CoherOF (*t*(24) = −5.61, *p* < 0.001, Cohen's *d* = −0.57), TempOF (*t*(24) = −4.34, *p* < 0.001, Cohen's *d* = −0.44), or Static (*t*(24) = −8.73, *p* < 0.001, Cohen's *d* = −0.89), as well as lower for NormalBM than CoherOF (*t*(24) = −4.62, *p* < 0.001, Cohen's *d* = −0.47) or Static (*t*(24) = −7.73, *p* < 0.001, Cohen's *d* = −0.788) but not for TempOF (*p* = 0.05). There was also no significant difference between the bCFS values for CoherOF and TempOF. However, TempOF had a significantly lower bCFS than Static (*t*(24) = −4.39, *p* < 0.001, Cohen's *d* = −0.45).

Motion types also differed in reCFS values. In particular, reCFS thresholds for the Static condition were significantly higher than all other conditions (FastBM: *t*(24) = −7.61, *p* < 0.001, Cohen's *d* = −0.78; NormalBM: *t*(24) = −9.85, *p* < 0.001, Cohen's *d* = −1.00; CoherOF: *t*(24) = −5.92, *p* < 0.001, Cohen's *d* = −0.60; and TempOF (*t*(24) = −7.525, *p* < 0.001, Cohen's *d* = −0.77). Additionally, NormalBM had a lower reCFS threshold than CoherOF (*t*(24) = −3.931, *p* = 0.006, Cohen's *d* = −0.40) but was not significantly different from TempOF. There were no other significant differences in reCFS documented; namely, there was no significant difference between the reCFS value for FastBM and NormalBM. In summary, unlike the bCFS measures, all motion types had reCFS values significantly lower than the mean reCFS for Static.

### Experiment 3 discussion

Experiment 3 demonstrated an effect of motion type on suppression depth, in contrast with previous tCFS literature that found a consistent suppression depth between different static image categories (Alais et al., 2024). Specifically, we found a significant reduction in suppression depth for fast biological motion and a static biomotion figure, relative to normal-speed biomotion, and both coherent and temporally scrambled optic flow. The difference between a static figure and the movement conditions suggests that there is differential processing of motion information into conscious awareness when compared to static imagery (Chung & Khuu, 2014; Moors, Wagemans, & de-Wit, 2014; Perrone, 2004), which may underpin why we see changes in suppression depths not previously found with various static imagery (Alais et al., 2024).

One interpretation of this result is that the motion in these stimuli makes them harder to suppress, likely as the motion contributes a resistance to adaptation, which enhances the perceptual stability of the targets (Blake, Yu, Lokey, & Norman, 1998). Neurophysiological studies also suggest that motion stimuli first enter V1 with a “magnocellular advantage” of 20 to 30 ms (Bullier, Hupé, James, & Girard, 1996; Laycock, Crewther, & Crewther, 2007), which could contribute to asymmetries in interocular suppression. Together, this indicates an earlier (and broader) V1 activation pattern by these motion stimuli, which may partially explain their earlier access into awareness (bCFS) and maintained suppression (i.e., suppression depth) relative to the static stimuli.

Importantly, even though the global motion types differed, our normal biomotion and optic flow conditions were programmed with similar dot speeds. Therefore, the similarity of suppression depth between these conditions may highlight the important contribution of local motion information to suppression depth. The global integration of biological form from motion (Johansson, 1973) provides no advantage to conscious awareness when measured with suppression depth, as no difference was observed when comparing normal biological motion with radial and temporally scrambled optic flow motion. Alongside the lack of an inversion effect identified in Experiments 1 and 2, this result provides further evidence for the local-precedence hypothesis (Sun et al., 2022).

Looking specifically at variations in biological motion speed, curiously, the normal speed and the fast walker had a similar decrease in bCFS when compared to the static figure. This suggests that bCFS is conserved at the same contrast threshold for biological motion at two speeds. This is surprising, as we would expect higher speeds of motion to lead to greater resistance to neural adaptation (Blake et al., 1998; Moors et al., 2014), where faster targets should break through at lower thresholds, as has been shown previously (Ananyev et al., 2017). Notably, however, Ananyev and colleagues (2017) used a colorful rotating mask type that is distinct from our own, and different mask parameters likely influence suppression and perceptual stability in CFS, where perceptual stability of motion seems to be dependent on a distinct set of parameters organized by the visual pathway (rather than a feature-selective process, as seen with color and shapes; Breitmeyer et al., 2006; Stuit, Cass, Paffen, & Alais, 2009).

This is mirrored by others who have shown that bCFS peaks when using masks refreshing at a low temporal frequency (Han, Lunghi, & Alais, 2016). With bCFS shown to also peak at high frequency, this was taken as evidence that suppression in CFS has a parvocellular bias. However, in the context of motion, where magnocellular neurons are tuned to high temporal and low spatial frequencies, this unique interplay between mask and target speed is not well understood. Here, others have highlighted that a regular CFS mask used for static targets may not be best optimized for the suppression of moving targets (Moors et al., 2014). Therefore, further psychophysical and imaging studies are required to address our null results for bCFS with increased biomotion speeds.

Focussing now specifically on optic flow motion, rejecting our hypothesis, we did not replicate the results of Chung and Khuu (2014) and Kaunitz et al. (2013), who identified that coherent motion imagery had lower bCFS than random trajectory motion. Here, we found that presenting a coherent or temporally scrambled optic flow does not alter bCFS or suppression depth. This suggests that, in our case, the coherent global configuration of the motion does not have an impact on the mechanism of suppression.

We also observed a remarkably conserved mean suppression depth for the walker viewed at 90° (13.2 dB), in line with the same walker oriented toward the viewer, as per Experiment 1 (UprightBM = 13.2 dB) and Experiment 2 (UprightBM, medium mask contrast = 13.1 dB). Even though these experiments were performed with different participants, these results allow us to be confident that the viewing angle and orientation of the walker were not a confound that provided unique results. Together, these experiments provide complementary evidence that walking orientation or rotation does not have a significant influence on both bCFS and suppression depth, as opposed to the biomotion inversion effect suggested by others (Sun et al., 2022; Watson et al., 2004).

## General discussion

We performed three experiments to assess the relative contributions of low- and high-level features of biological motion to interocular suppression depth, using the new tCFS procedure (Alais et al., 2024). Experiment 1 demonstrated no change in suppression depth for the inversion of biological motion, which we replicated in Experiment 2. Experiment 3 extended this result by demonstrating that manipulating local dot speed while keeping global form constant decreased suppression depth, while maintaining local dot speed and varying global form had no effect on suppression depth. Together, this highlights the importance of local motions rather than global motion (and global form *from* biological motion) in CFS.

The cumulative findings of our three experiments highlight that variations in suppression depth can occur with variant forms of motion. Previously, tCFS had only been used with static images, and uniform suppression depth was reported (Alais et al., 2024). Overall, our data support the suppression mechanism's dependency on local form processing, further supporting the local-precedence hypothesis of interocular suppression (Sun et al., 2022), whereby the suppression mechanism operates in local spatial zones (i.e., Han et al., 2019) and is influenced by local features (e.g., speed, spatial configuration, local motion type). To discuss this further, a few patterns of results are important to emphasize.

In our data, no significant difference in either bCFS or reCFS thresholds was found between upright and inverted stimuli across Experiments 1 and 2, suggesting that the global upright biomotion form did not asymmetrically influence access into awareness. We replicated this lack of an inversion effect and found it maintained across three levels of mask contrast (Experiment 2) and additionally found that horizontal rotation of the biomotion figure resulted in highly similar suppression depths across unique groups of participants (Experiment 3).

This pattern of results—wherein global form did not alter suppression—is in contrast to the stated biological motion inversion effect (Sun et al., 2022; Watson et al., 2004). Specifically, biological motion inversion was found to significantly lower detection rates (relative to upright biological motion) during a bCFS procedure that measured breakthrough times (Sun et al., 2022). A similar advantage occurs during binocular rivalry, wherein viewing upright biological motion results in greater periods of monocular dominance compared to inverted figures (Watson et al., 2004). This discrepancy between our present and these past results may partly be explained by differences in experimental methods. Sun and colleagues (2022) used a short-interval two-alternative forced-choice (2AFC) procedure in which suppressed targets increased in contrast for the first 900 ms of 2,000-ms trials. The abrupt change and maintenance of contrast could lead to patterns of breakthrough that are distinct from the tCFS procedure, where contrast does not reach a sustained high-contrast phase. Watson et al. (2004) deployed binocular rivalry to evidence an inversion effect, which, though similar to CFS, has been shown to be susceptible to potential attentional effects (Blake, Goodman, Tomarken, & Kim, 2019; Chong & Blake, 2006) and exerts less suppression on visual stimuli in early cortex than CFS (Yuval-Greenberg & Heeger, 2013), and thus local signals are more likely to flow to subsequent global processing areas, where an inversion effect is expected and may in turn exert feedback to earlier visual areas (van Boxtel, Alais, & van Ee, 2008). More specifically, it is possible that the inversion effect reported during rivalry is partially mediated by attention or an increase in saliency that is absent during CFS. There is some previous evidence that higher-level processing does occur without awareness in the context of coherent motion (Chung & Khuu, 2014; Kaunitz et al., 2013). Reports using fMRI have also shown that when motion is suppressed from conscious awareness, it is processed by areas of the dorsal stream, particularly the human Middle Temporal area + (hMT+) region, and this does not occur when viewing static imagery (Alexander & Cowey, 2009; Tong, Meng, & Blake, 2006). Thus, the processing of global form outside of awareness may be possible, yet it is not supported by a biological motion inversion effect in our data.

Others, however, have suggested that motion processing outside of awareness is based on local motion detectors and therefore confined to simple translational motion, a process that the V1 is capable of encoding (Maruya et al., 2008). This process is therefore not dependent on the binding of spatiotemporal information in higher areas such as the medial temporal/medial superior temporal (MT/MST) regions, helping to explain the presence of motion aftereffects of suppressed motion imagery during interocular rivalry (Kaunitz et al., 2011; Maruya et al., 2008). This places motion coherence with other low-level features that are processed in the absence of conscious awareness (Ananyev et al., 2017; Blake et al., 1998). This would also clarify why others found no difference between biological motion and a biological random trajectory control, where sinusoidal patterns and local motion information were intact (Kaunitz et al., 2013).

Neurophysiological studies also suggest that motion and form are processed at different speeds before being combined (Boussaoud, Ungerleider, & Desimone, 1990). For example, motion information from the dorsal stream (namely from the MST) arrives at the posterior superior temporal sulcus (pSTS) some 20 ms before form information is received from the ventral stream (namely the anterior inferior-temporal area; Boussaoud et al., 1990). Indeed, the binding of both the biological object's motion and form has been shown to occur 100 ms after the visual stimulus has become visible (Oram & Perrett, 1996). In line with our results, this suggests that the suppression mechanism is facilitated within earlier visual processing areas well before motion information is bound with biological form and is therefore independent of any associated feedback mechanisms (i.e., from the pSTS; Grossman et al., 2000).

This evidence of a low-level mechanism is also supported by previous research that manipulated rates of adaptation during tCFS. Alais et al. (2024) manipulated the rate of contrast change for static targets and found that increased rates led to an increased suppression depth (Alais et al., 2024), indicating that suppression is deeper when there is less time for adaptation. Evidence for low-level competition is also present during the fractionation of images during CFS (Moors et al., 2017; Zadbood, Lee, & Blake, 2011), wherein patches, features, or piecemeal components of suppressed stimuli become visible, highlighting the presence of interocular competition within relatively small receptive fields. The present work extends this line of evidence to include motion stimuli and indicates that the local low-level processing of dot motion is the likely contributor to coherent motion's priority access to conscious awareness (Chung & Khuu, 2014; Kaunitz et al., 2013).

It is worth noting that in Experiment 3, our results did not show a difference between coherent and temporally scrambled optic flow bCFS values, which contrasts with similar prior research (e.g., Kaunitz et al., 2013). Importantly, Kaunitz et al. (2013) found that coherent radial motion was more detectable than random-dot motion, yet important differences exist between our stimuli. Their coherent motion stimuli were made of dots moving toward a center point, local speed did not vary relative to the center, and dots were redrawn at a random location after arriving at the center point. In comparison, our optic flow stimuli did vary in local speed with the distance from the center via a sinusoidal expansion and compression. It is plausible that our coherent motion conditions, intended as a control, perhaps involved an insufficient alteration to influence bCFS, reCFS, or suppression depth measures. Indeed, sinusoidal motion has been previously rated by subjects as “biological-like” (Chang & Troje, 2009), suggesting future research may benefit from more artificial motion types as control conditions.

## Future directions

Therefore, future studies will benefit from a nonsinusoidal control in each experiment to identify the contributions of biomotion and biomotion-like sinusoidal specific influences (e.g., Fleishman & Pallus, 2010). For example, an appropriate control could include a triangle wave pattern with the same dot trajectories and an average speed based on each dot from the biological motion (Chang & Troje, 2009; Sun et al., 2022). Indeed, this has been used by others who identified a significant difference in bCFS between upright biomotion and their non-biomotion control (Sun et al., 2022). Furthermore, investigating increases/decreases in the speed of different target types (alongside different moving mask types; e.g., Moors et al., 2014) will further outline the unique interplay between mask and target, as well as the local feature requirements responsible for organizing suppression and priority access to conscious visual perception.

## Conclusions

Continuing the line of inquiry from previous studies utilizing BR and bCFS methodologies, this research employed the novel tCFS method to examine the impact of motion type on suppression thresholds and depth of suppression in CFS. Here, the cumulative findings of our three experiments highlight that variations in suppression depth can occur with different forms of motion. Furthermore, biological inversion, optic flow transformations, rotation, and various global motion scrambling did not influence suppression depth, but changing the spatial extent of stimuli and local motion speed both altered suppression depth. Overall, our findings support the local-precedence hypothesis of interocular suppression, in contrast to previous research suggesting biological or coherent form is processed outside of awareness.
